# The in vitro effects of probiotic bacteria on genital pathogens of female dogs

**DOI:** 10.1186/s12917-023-03635-y

**Published:** 2023-07-08

**Authors:** Golińska Edyta, Sowińska Natalia, Szydło Marlena, Witka Natalia, Lenarczyk Joanna, Zbigniew Arent, Strus Magdalena

**Affiliations:** 1 Chair of Microbiology, Jagiellonian University Medical College, Czysta 18, 31-121 Cracow, Poland; 2 Animal Reproduction Unit, Department of Genetics and Animal Breeding, Faculty of Veterinary Medicine and Animal Science, Poznan University of Life Sciences (PULS), Wojska Polskiego 28, 60-637 Poznań, Poland; 3 Veterinary Clinic Biały Kieł, Białoruska 17a, 30-638 Cracow, Poland; 4 Veterinary Clinic Felis, Dworcowa 32, 32-620 Brzeszcze, Poland; 5 Veterinary Clinic Multivet, Feliksa Konecznego 6/12U, 31-216 Cracow, Poland; 6 University Center of Veterinary Medicine of the Jagiellonian University and Agricultural University of Cracow, Center of Experimental and Innovative Medicine, University of Agriculture in Cracow, Al. Mickiewicza 24/28, 30-059 Cracow, Poland

**Keywords:** Antibacterial activity, Vaginal microflora, Dog, Lactobacillus, Probiotics

## Abstract

**Background:**

An important aspect in the microbiology of the reproductive system of small animals is the potential occurrence of probiotic bacteria, such as lactic acid bacteria (LAB) of the genus *Lactobacillus*. The presence of these microorganisms is significant due to their strong antibacterial and antifungal properties. This study aimed to select probiotic strains from the oral cavity and vagina that have outstanding antibacterial properties against typical genital pathogens of the female dog reproductive tract.

**Results:**

The antagonistic activity of ten LAB strains was tested against seven etiological agents isolated from the genital tract of female dogs with signs of inflammation. LAB strains with the greatest ability to inhibit the growth of indicator bacteria were *Lactobacillus plantarum* and *L. acidophilus*, while *L. fermentum* and *L. brevis* strains inhibited growth the least. Almost all strains showed a complete lack of adherence to Caco-2 epithelial cells.

**Conclusions:**

All tested LAB isolates inhibited the in vitro growth of either Gram-positive or Gram-negative pathogens, suggesting that potential probiotic strains could contribute to the balance of the normal vaginal microbiota. Furthermore, they could be considered for use as prophylactic agents or as an alternative to antibiotic therapy for infections in dogs.

## Background

Numerous studies defining vaginal microflora in dogs do not consider strains of the genus *Lactobacillus* spp*.*, with Hutchins et al. [1] reporting a lack of this population in the microbiota, which correlates with the results of our research [2]. Indeed, these strains are not evident in the vaginal environment due to the typically high pH of between 6.5 and 7.5 [2, 3]. For comparison, the pH in the human female vagina is 4.5 or less [3], and lactic bacteria grow in the range of 4.5 to 7.0, with optimum growth at pH 6.0, while species such as *L. plantarum* grow at a pH ranging from 4.0 to 8.0 [4]. Nonetheless, the first reports on isolating *Lactobacillus* spp*.* from female dogs appeared in 2008 [3].

Probiotics are defined by the World Health Organization (WHO) as live microorganisms that have a beneficial effect on host health when given in adequate amounts (Food and Agriculture Organization of the United Nations and WHO Working Group, 2002). Probiotic bacteria include, among others, three types of lactic acid bacteria (LAB) from the genera *Lactobacillus, Bacillus,* and *Bifidobacterium*, and colonize the oral cavity, digestive tract, and vagina of most mammals. The antibacterial potential of LAB is due to the production of lactic acid, which results in the acidification of the environment and the formation of bacteriocins and hydrogen peroxide [1]. An effective probiotic is characterized by its potential for host colonization, its ability to interfere with colonization by pathogens, and its adherence to epithelial cells. Meanwhile, these strains should not have pathogenic potential and should be resistant to gastric juices and bile salts [3, 5].

Studies performed on the LAB bacteria of females showed that they regulate the microflora of the urogenital tract and have antagonistic properties against pathogenic bacteria. Since *Lactobacillus* strains constitute a physiological component of the female genital tract, this research attempted to isolate probiotic bacteria from the dog vaginal tract.

The first study on probiotic bacteria in the genital tracts of healthy female dogs was conducted by Delucchi et al*.* [3], who demonstrated the presence of LAB in the vagina, with *Lactobacillus* isolated in 59% of cases (out of 42 examined dogs). The isolated strains inhibited the in vitro growth of pathogenic bacteria, including *Escherichia coli, Proteus mirabilis,* and *Staphylococcus aureus*, suggesting that LAB plays a beneficial and protective role in infections of the female urogenital tract. Although the research described above shows that LAB may be a component of the vaginal microflora, their ability to adhere to vaginal epithelium has not been demonstrated. The most abundant vaginal bacteria in dogs derive from *Lactobacillus* and *Enterococcus* species [3, 6], with *L. gasseri, L. brevis,* and *L. acidophilus* showing excellent adherence to vaginal epithelial cells [7–9]. However, they do not have such effective properties in dogs.

This study aimed to select probiotic strains of bacteria isolated from the oral cavity and vagina that have outstanding antibacterial properties against typical genital pathogens of the female dog reproductive tract.

## Methods

### Microbiological culture

Client-owned dogs presented to the Veterinary Clinic of the University Center of Veterinary Medicine of the Jagiellonian University and Agricultural University of Cracow (JU-AU), Poland, in connection with reproductive problems, estrous monitoring, determination of the mating date, or routine gynecological examination of breeding dogs. The owners were informed about the purpose of the study and gave their written consent for their dogs to participate.

Sampling for microbiological examination, cytological smears, serum progesterone level determination, and clinical group stratification was performed as previously described [2]. The samples for microbiological examination were taken from the dorsal section of the upper vaginal vault using a sterile swab and a sterile Hannover vaginal specula (Eickemeyer, Tuttlingen, Germany) of 150 mm in length and 5, 10, or 15 mm diameter (size adjusted to the bitch). Additionally, samples were taken from the oral cavity using a sterile swab. All microbiological samples were delivered to the laboratory in Amies transport medium (Deltalab, Barcelona, Spain) within four hours of collection.

The swab was transferred from the transport medium to 1 ml of Schaedler broth (Becton, Dickinson and Company, MD, USA) and agitated for 1 min [2]. Serial decimal dilutions were then made in the same broth, and 100 μl aliquots were plated on standard media for cultivation, including McConkey agar (Oxoid Ltd, Hampshire, UK) for *Enterobacteriaceae*, Columbia blood agar with 5% sheep blood (Oxoid) for Streptococci, bile esculin azide (BBL™) Enterococcosel™ agar (Beckton Dickinson, NJ, USA) for enterococci, Rogosa Agar (Merck, Darmstadt, Germany) for *Lactobacilli*, and Saboraud Agar (Merck) for *Candida* spp. The dilutions were then spread over the plate surface by a glass rod, and plates were incubated at 35 °C for 24 h (for aerobic bacteria) or 48 h under microaerophilic conditions (for *Lactobacilli*) [2]. The morphology of the grown colonies was assessed using a magnifying glass, and several colony picks of each morphological type were subcultured on appropriate media and Gram-stained. After making subcultures, all colonies representing different morphotypes were counted on the plates showing appropriate colony density. Bacterial numbers were expressed as the log10 of colony forming unit per 1 ml (CFU/ml) [2]. The subcultured colonies were further incubated, and after checking for purity of the cultures, phenotypic identification was performed using commercial identification systems, analytical profile index (API) 20E, API50CH, APIStaph, APIStrep, and API20NE (bioMerieux, l’Etoile, France).

When API tests were inconclusive, polymerase chain reaction (PCR) was performed with species-specific primers Llac-f 5’-GGCGGCTTACTGGACAAC-3’ and Llac-r 5’-CTTAGACGGCTCCTTCCAT-3’ for *L. lactis*, and LcR-F 5’-CGTTGCATAGAGTGGAAAATTATG-3’ and Lc-R 5’-GTTGAGCCACTGCCTTTTAC-3’ for *L. rafinolactis*. Amplification was performed according to the methodology described by Lee and Odamaki [10, 11].

### Typical pathogenic bacteria isolated from the vagina (indicator bacteria)

Indicator bacteria were those isolated from female canines aged six months to ten years (from our earlier publications) [2] with typical signs of inflammation of the genital tract (mucous, milky-white, greenish or yellowish vaginal discharge, with the cytology of the vaginal epithelium containing numerous neutrophils, mucus bands and fragments of epithelial cells). The isolates were used in the experiments to evaluate the antibacterial activity of LAB.

### Probiotic bacteria

Bacteria of the genera *Lactobacillus, Leuconostoc*, *and Lactococcus* (n = 10) were isolated from the oral cavity of four-year-old female dogs (n = 5). These bacteria have been subjected to in vitro tests to determine their possible probiotic properties, paying particular attention to their antagonistic properties against indicator bacteria and their adhesion to intestinal epithelial cells.

### Semi-quantitative agar slab method

The agar slab method is used for the semi-quantitative evaluation of the activity of probiotic bacteria on a population of indicator bacteria. The antagonistic activity of ten strains of LAB was tested against seven selected etiological agents isolated from the genital tract of female canines with signs of inflammation using the agar slab method of Strus et al. [12, 13]. A suspension of *Lactobacillus* strains with a density of two on the McFarland scale (according to McFarland Standards) was plated on MRS (de Man, Rogosa, and Sharpe) medium and incubated at 37˚C for 48 h under anaerobic conditions. Subsequently, disks with a 9 mm diameter were cut from the solid medium using a sterile cork borer and placed on an appropriate agar medium containing strains of tested pathogens at a density of 0.5 on the McFarland scale. Further cultivation was carried out under aerobic conditions at 37˚C for 24 h. Then, the diameter of the growth inhibition zone around the agar slabs containing probiotic strains was measured.

### Quantitative method

The quantitative method allowed for more accurate observations of the kinetics of the interaction of LAB strains with the pathogenic bacterial population. LAB strains were propagated in MRS liquid medium under anaerobic conditions at 37° C for 72 h, with a density of approximately 5 × 10^7^ CFU/ml achieved. Indicator bacteria were suspended in sterile physiological saline to obtain a concentration of around 1 × 10^8^ CFU/ml. Each cultured probiotic strain was placed in a sterile tube (900 μl), and the indicator bacteria suspension was added to each (100 μl). The probiotic and indicator bacteria mixtures were plated in dilutions after 0.8 and 24 h and incubated at 37 °C aerobically for 24 h. All indicator colonies were counted on the plates with sufficient colony density.

### Adherence properties of canine lactic acid bacteria strains

The ability of the *Lactobacilli* strains to adhere to the gut epithelium was assessed in vitro using the Caco-2 human colon carcinoma epithelial cell line (American Type Culture Collection, Middlesex, United Kingdom). The cell line is the best-described and characterized intestinal cell line representing mammalian intestinal lineage.

The cells were grown to confluence in Dulbecco’s Modified Eagle medium (DMEM)/F-12 (GIBCO, CA, USA), supplemented with 10% heat-inactivated fetal bovine serum (FBS) (GIBCO), on 0.4 µm semipermeable Transwell™ tissue culture inserts (Corning, NY, USA) in a humidified incubator at 37 °C and 5% CO^2^. Briefly, 48-h cultures of Caco-2 cells at a density of 1 × 10^6^ cells/ml were incubated for 24 h in 12-well flat bottom tissue culture plates (Iwaki, Fukushima, Japan) in Eagle’s 1959 medium (MEM) (Biomed, Lublin, Poland) containing L-glutamine, sodium bicarbonate (NaHCO^3^) (IITD, Wrocław, Poland), 5% fetal calf serum (Sigma-Aldrich Chemie, Germany), and antibiotics (penicillin 100UI/ml, streptomycin 100UI/ml, neomycin 200 μg/ml) (Sigma Aldrich, Chemie, Germany), then washed twice with phosphate-buffered saline (PBS).

Overnight bacteria cultures were diluted with MRS and MEM to a concentration of approximately 10^8^ CFU/ml and used to inoculate the plated cells. After incubation at 37 °C for 30 min, wells were washed twice with PBS to release unbound bacteria. Then, the cells were fixed with 3.7% formaldehyde for one hour, washed twice with PBS, and stained with crystal violet stain (Merck, Darmstadt, Germany). The adherent bacterial cells were counted in five randomly selected microscopic fields, and the degree of adhesion was evaluated using a semi-quantitative scoring system ranging from 0 to 3 based on the following characteristics:

- strong adherence (3): > 80 bacterial cells per field
- moderate adherence (2): 61 - 80 bacterial cells per field
- weak adherence (1): 41 - 60 bacterial cells per field
- no adherence (0): < 40 bacterial cells per field

All experiments were run in duplicate.

### Statistical analysis

The statistical significance of differences between antagonistic properties of LAB strains was analyzed using a one-way analysis of variance (ANOVA). The significance level was set at *P* < 0.05.

## Results

This research used seven strains of pathogenic bacteria that cause inflammation of the dog reproductive tract as indicators, based on previous work by Golińska et al. [2]. The strains belong to five species: *E. coli* (two strains), *Staphylococcus intermedius* (two strains), *Klebsiella pneumoniae, Enterococcus faecalis,* and *Streptococcus canis* (Table 1).

**Table 1:** Characteristics of the indicator bacteria used in the study

No	Indicator bacteria strain	CFU/ml	Age of dog (years)	Vaginal pH	Estrous phase	Clinical group
1	*Escherichia coli* P37	1.0E + 04	**4**	6.9	Anestrus	With genital tract infections
2	*Escherichia coli* P39	3.0E + 03	**4**	6.6	Anestrus	With genital tract infections
3	*Klebsiella pneumoniae* P41	1.0E + 02	**4**	7.2	Anestrus	With genital tract infections
4	*Enterococcus faecalis* P37	3.0E + 02	**4**	6.9	Anestrus	With genital tract infections
5	*Streptococcus canis* P37	1.0E + 04	**4**	6.9	Anestrus	With genital tract infections
6	*Staphylococcus pseudintermedius* P40	1.0E + 03	**1**	6.0	Anestrus	With genital tract infections
7	*Staphylococcus intermedius* P41	1.0E + 04	**4**	6.9	Anestrus	With genital tract infections

LAB strains isolated from all tested samples originating from the oral cavity of dogs included ten strains belonging to eight species: *Lactobacillus acidophilus* (two strains), *Lactobacillus fermentum* (two strains), *Lactobacillus plantarum*, *Lactobacillus paracasei, Lactobacillus crispatus, Lactobacillus brevis, Lactococcus raffinolactis*, and *Leuconostoc lactis* (Table 2). The vaginal swabs contained no LAB.

**Table 2:** Characteristics of the lactic acid bacteria used in the study

No	Lactic acid bacteria	CFU/ml	Age of dog (years)	Place of isolation
**1**	*Lactobacillus plantarum/1*	2.80E + 05	4	oral cavity
**2**	*Lactobacillus paracasei/2*	3.70E + 05	4	oral cavity
**3**	*Lactobacillus acidophilus/3*	1.00E + 05	4	oral cavity
**4**	*Lactobacillus fermentum/4*	7.90E + 05	4	oral cavity
**5**	*Lactobacillus crispatus/5*	2.80E + 04	4	oral cavity
**6**	*Lactobacillus acidophilus/6*	1.00E + 06	4	oral cavity
**7**	*Lactobacillus fermentum/7*	4.90E + 04	4	oral cavity
**8**	*Lactococcus raffinolactis/8*	5.00E + 05	4	oral cavity
**9**	*Leuconostoc lactis/9*	3.10E + 05	4	oral cavity
**10**	*Lactobacillus brevis/10*	2.80E + 05	4	oral cavity

**Fig. 1:**
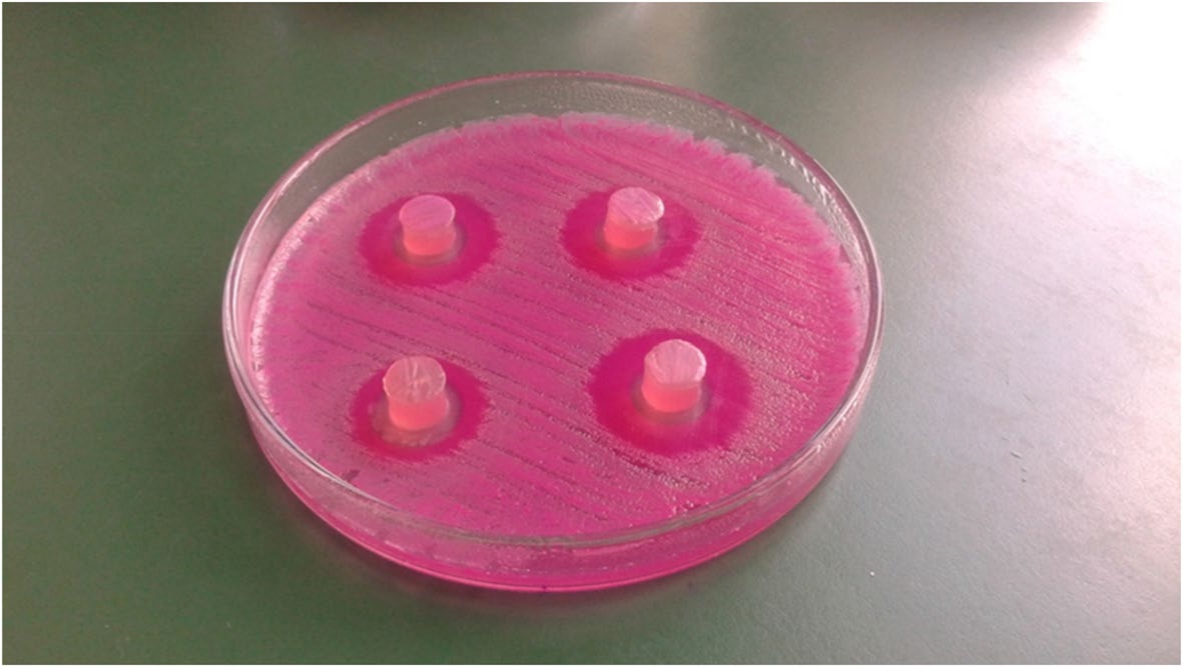
A sample photograph from the semi-quantitative study on the antagonistic abilities of selected strains with probiotic properties against* Escherichia coli*

The antagonistic activity of ten strains of LAB was tested against seven selected etiological agents isolated from the genital tract of canines with signs of inflammation, and the results are presented in Fig. 1 and Table 3. The diameter of the growth inhibition zones of the test bacteria induced by the LAB ranged from 9 to 21 mm, and the slab diameter was 9 mm. In general, the greatest inhibition was observed for *E. coli* P37 and *S. canis* P37, while the smallest was found for *S. pseudintermedius* P40 and *S. pseudintermedius* P41. The LAB strains showing the greatest ability to inhibit the growth of indicator bacteria were *L. plantarum* and *L. acidophilus*. Meanwhile, the smallest growth inhibition was induced by the *L. fermentum* and *L. brevis* strains.

**Table 3:** The effect of different lactic acid bacteria strains on the growth of indicator bacteria, as determined by the agar slab method. Zones of growth inhibition are shown in millimeters (mm)

		**Indicator bacteria (zones of growth inhibition in millimetres)**						
**No**	***Lactic acid bacteria***	***E. coli P37***	***E. coli P39***	***K. pneumoniae P40***	***E. faecalis P37***	***S.canis P37***	***S.intermedius P13***	***S.intermedius P40***
**1**	***Lactobacillus plantarum/1***	21	15	15	12	20	11	11
**2**	***Lactobacillus paracasei/2***	16	12	9	9	16	9	9
**3**	***Lactobacillus acidophilus/3***	21	17	16	15	20	12	13
**4**	***Lactobacillus fermentum/4***	14	13	9	9	14	9	9
**5**	***Lactobacillus crispatus/5***	18	15	14	13	19	13	12
**6**	***Lactobacillus acidophilus/6***	19	16	13	10	20	12	12
**7**	***Lactobacillus fermentum/7***	14	13	9	9	14	9	9
**8**	***Lactococcus raffinolactis/8***	18	17	13	12	20	12	10
**9**	***Leuconostoc lactis/9***	18	17	13	12	20	11	11
**10**	***Lactobacillus brevis/10***	15	12	9	9	14	9	9

The five strains of LAB that induced the greatest growth inhibition of the indicator bacteria in the semi-quantitative study were selected for the antagonism studies using the quantitative method. These strains were: *L.plantarum*/1, *L. acidophilus*/3*, L.acidophilus*/6, *Leuconostoc lactis*/9, and *Lactococcus raffinolactis*/8.

**Fig. 2:**
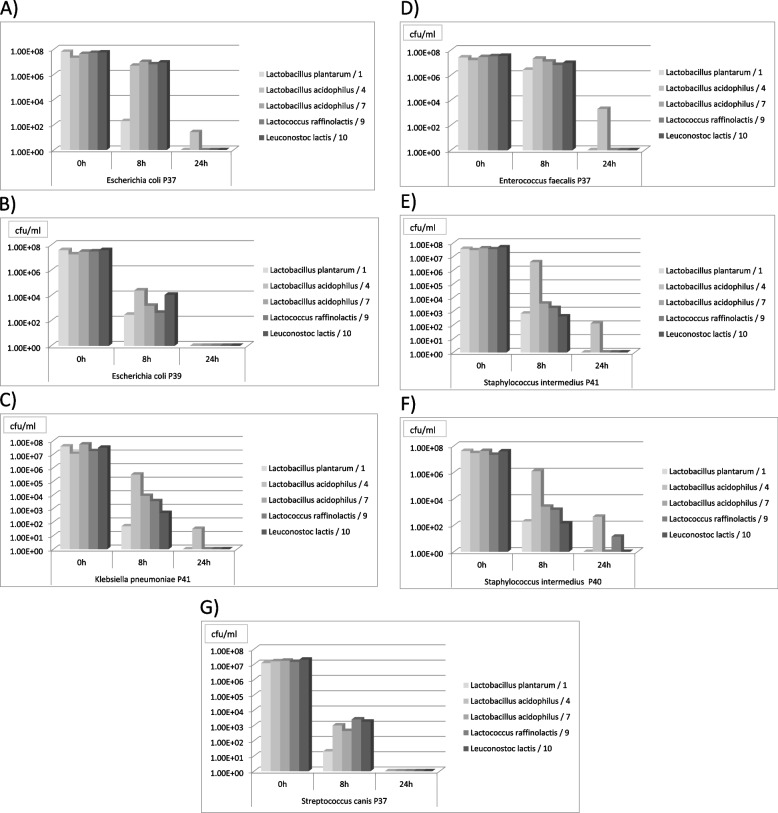
The antagonistic effect of selected strains with probiotic properties against indicator bacteria (quantitative method)

The greatest antagonistic activity was shown for *L. plantarum*/1, which reduced the concentration of *K. pneumoniae* and *S. canis* from 10^7^ CFU/ml to 10^1^ CFU/ml within eight hours, and both of the *E. coli* and *S. pseudintermedius* strains from 10^7^ CFU/ml to 10^2^ CFU/ml. However, it was least effective against *E. faecalis* P37, whose CFU/ml only decreased by one log after eight hours of the experiment. Nonetheless, none of the indicator bacteria strains grew after incubation with *L.plantarum*/1 for 24 h. The *L. acidophilus* /6 and *L. lactis* /9 strains also showed complete inhibition of the growth of indicator bacteria after 24 h of cultivation. The weakest antagonistic effect was observed for the *L. acidophilus*/3 strain, although statistical analysis showed no correlation between specific LAB strains and antagonistic properties against any indicator bacteria. All results are presented in Fig. 2.

**Fig. 3:**
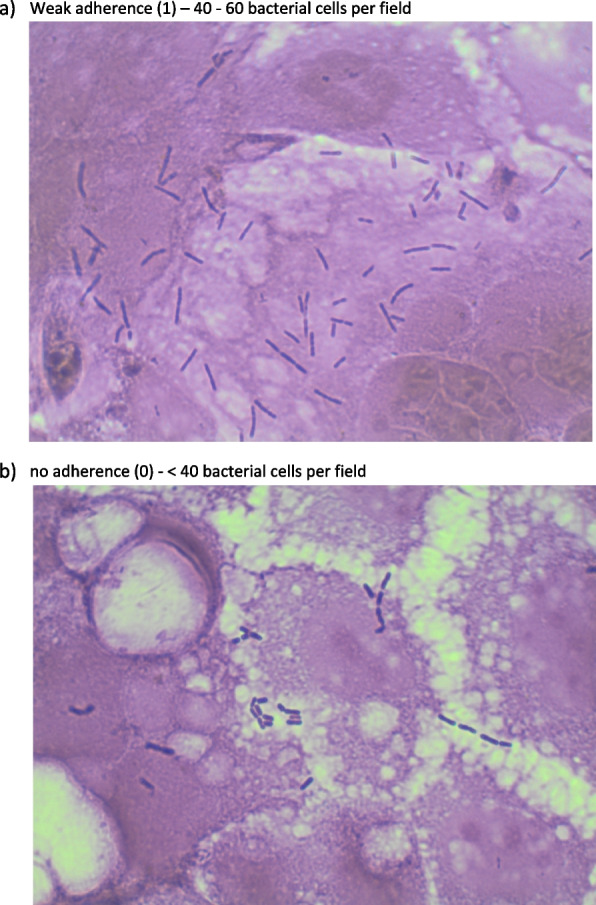
Sample photographs of bacterial adherence

An adherence assay was performed on the five LAB strains that induced the greatest growth inhibition of the indicator bacteria during the semi-quantitative study. Only *Lactococcus raffinolactis* /8 demonstrated weak adherence, with the rest of the tested strains showing a complete lack of adherence to the Caco-2 epithelial cells (Fig. 3 and Table 4).

**Table 4:** Degree of bacterial adhesion to Caco-2 tissue

**Probiotic strains**	**Numbers of cells in 1st field of viev**	**Numbers of cells in 2nd field of viev**	**Numbers of cells in 3rd field of viev**	**Numbers of cells in 4th field of viev**	**Numbers of cells in 5th field of viev**	**Average number of cells in the field of view**
***Lactobacillus plantarum/1***	30	20	20	20	15	**21**
***Lactobacillus acidophilus/4***	10	10	10	8	5	**8,6**
***Lactobacillus acidophilus/7***	30	20	30	30	30	**28**
***Lactococcus rafinolactis/9***	50	40	5	50	40	**46**
***Leuconostoc lactis/10***	20	30	15	20	20	**21**

## Discussion

Inflammatory diseases of the female dog reproductive tract are a common problem in veterinary practice. The inflammation can lead to serious health problems, among which the most important are fertility disorders, embryo resorption and mortality, endometritis-pyometra syndrome, and urinary system disorders. Most genital tract infections in bitches are of bacterial origin since the vagina and uterus are rich environments for bacteria to live in.

The physiological vaginal microflora is formed of saprophytic and conditionally pathogenic bacteria, most of which are aerobic, as described in our earlier work [2] and the works of other authors [1]. Our previous results [2] suggest that the quantity and type of bacteria vary between different stages of the estrous cycle, and the total number of bacteria was significantly higher in healthy dogs during the proestrus/estrus and diestrus phase than in the anestrus phase, though there were no significant differences in the number of bacteria between healthy dogs and those with vaginitis. Moreover, the prevalence of common pathogens, such as *E. coli*, *S. pseudintermedius*, *S. canis*, and *Enterococcus* spp., was similar between healthy dogs and those with genital tract infections. However, the studies carried out so far do not demonstrate which pathological flora are physiological, which prompted the search for alternative methods of treatment or prevention of bacterial infections of the genital tract in an era of increasing antibiotic resistance in dogs.

Studies performed on females showed that their LAB have antagonistic properties against pathogenic bacteria and can regulate the urogenital tract microflora [14–16]. Moreover, the probiotics *L. gasseri*, *L. brevis*, and *L. acidophilus* are known to have an excellent degree of adherence to vaginal epithelial cells in females [17, 18]. However, no probiotic bacteria have been used to treat genital tract infections in bitches to date, and we believe that defining such bacteria is crucial if they are to be used to prevent bacterial inflammatory disorders.

The results of the agar slab study showed that dog-derived LAB have growth-inhibiting properties against vaginal bacterial pathogens and that the antagonistic effect depends on the type of pathogen. In general, the greatest inhibition was observed for *E. coli* and *S. canis* and the smallest for *S. pseudintermedius*. Meanwhile, the strongest antibacterial activity was found in the *L. acidophilus, L. plantarum, L. fermentum,* and *Lactococcus raffinolactis* species. These findings are in agreement with those of Delucchi et al. [3], who showed selected isolates to have antimicrobial activity against pathogenic bacteria. Moreover, Fraga et al. [19] and Dec et al. [20] observed a greater antibacterial effect of chicken and mare *lactobacilli* against bacterial pathogens in vitro.

Antimicrobial activity against common vaginal and urinary pathogens could be explained by diffusible substances produced by this bacterial group [21, 22]. All isolates assayed inhibited the in vitro growth of either Gram-positive or Gram-negative pathogens, suggesting that potential probiotic strains could contribute to the balance of the normal vaginal microbiota. Currently, animal probiotics are being increasingly used with great success in the treatment of intestinal diseases in dogs [23].

Adhesion to the mucosal epithelium is an important property for a strain to be considered a probiotic. In this study, only *Lactococcus raffinolactis* showed adhesion to Caco-2 tissue. This lack of adherence is confirmed by the research conducted by Hutchings et al. [6], where an oral probiotic supplement administered for two or four weeks did not increase the prevalence of vaginal LAB in dogs. Therefore, perhaps it is worth considering vaginal rather than oral probiotic delivery in such cases.

In summary, this study made it possible to select strains of LAB characterized by antagonistic properties towards bacterial pathogens resulting from the production of growth-inhibitory compounds and adhesive properties. Therefore, they can be considered for prophylactic use or as an alternative to antibiotic therapy for canine infections. However, it is possible that vagina-isolated LAB, such as *Enterococcus canintestini*, may provide improved colonization and adherence more often, though this requires further research.

## Data Availability

The data and materials are available from the corresponding author upon reasonable request.
